# Cervical lymph node metastasis classified as regional nodal staging in thoracic esophageal squamous cell carcinoma after radical esophagectomy and three-field lymph node dissection

**DOI:** 10.1186/1471-2482-14-110

**Published:** 2014-12-19

**Authors:** Junqiang Chen, Sangang Wu, Xiongwei Zheng, Jianji Pan, Kunshou Zhu, Yuanmei Chen, Jiancheng Li, Lianming Liao, Yu Lin, Zhongxing Liao

**Affiliations:** Department of Radiation Oncology, The Teaching Hospital of Fujian Medical University, Fujian Provincial Cancer Hospital, 91 Maluding, Fuma Road, Fuzhou, Fujian 350014 China; Xiamen Cancer Center, Department of Radiation Oncology, the First Affiliated Hospital of Xiamen University, Xiamen, 361003 China; Departments of Pathology, The Teaching Hospital of Fujian Medical University, Fujian Provincial Cancer Hospital, Fuzhou, 350014 China; Departments of Surgery, The Teaching Hospital of Fujian Medical University, Fujian Provincial Cancer Hospital, Fuzhou, 350014 China; Center of Oncology Research, Academy of Integrative Medicine, Fujian University of Traditional Chinese Medicine, Fuzhou, 350014 China; Department of Radiation Oncology, The University of Texas M. D. Anderson Cancer Center, Unit 97, 1515 Holcombe Boulevard, Houston, TX USA

**Keywords:** Esophageal cancer, Radiotherapy, Cervical lymph node metastasis, Prognosis, Tumor staging

## Abstract

**Background:**

Lymph node metastasis (LNM) is most common in esophageal squamous cell carcinoma (SCC). The bi-directional spread is a key feature of LNM in patients with thoracic esophageal SCC (TE-SCC). The purpose of this study was to analyze the prognostic factors of survival in patients with TE-SCC with cervical lymph node metastasis (CLM) and validate the staging system of the current American Joint Committee on Cancer (AJCC) in a cohort of Chinese patients.

**Methods:**

Of 1715 patients with TE-SCC who underwent radical esophagectomy plus three-field lymph node dissection at a single hospital between January 1993 and March 2007, 547 patients who had pathologically confirmed CLM (296 had surgery only and 251 had surgery + postoperative radiotherapy) were included in this study. The locations of the lymph nodes (LNs) were classified based on the guidelines of the Japanese Society for Esophageal Diseases.

**Results:**

The rate of CLM was 31.9% for all patients and was 44.2%, 31.5%, and 14.4% for patients with upper, middle, and lower TE-SCC, respectively (*P* < 0.0001). The rates of metastasis to 101 (paraesophageal lymph nodes), 104 (supraclavicular lymph nodes), 102 (deep cervical lymph nodes) and 103 (retropharyngeal lymph nodes) areas were 89.0%, 25.6%, 3.7% and 0.5%, respectively. The 5-year overall survival (OS) rate with CLM was 27.7% (median survival, 27.5 months). The 5-year OS rates were 21.3% versus 34.2% (median survival, 21.9 months versus 35.4 months) for after surgery only versus surgery + postoperative radiotherapy, respectively (*P* < 0.0001 for both). Multivariate analysis showed that the independent prognostic factors for survival were sex, pT stage, pN stage, number of fields with positive LNs, and treatment modality. In surgery only group, the 5-year OS rates were 24.1%, 16.2% and 11.7%, respectively, when there was metastasis to 101 LN alone, 104 LN alone or both 101 LN and 104 LN. The 5-year OS rates were 17.7%, 22.5% and 31.7%, for patients with upper, middle and lower TE-SCC , respectively (*P* = 0.112). The 5-year OS rates were 43.0%, 25.5%, 10.2% in patients with 1 field (cervical LNs), 2 fields (cervical + mediastinal, and/or cervical + abdominal LNs), and 3 fields (cervical + mediastinal + abdominal LNs) positive LNs, respectively (*P* < 0.0001). The number of fields of positive LNs did not impact the OS according to different pN stage (all *P* > 0.05).

**Conclusion:**

Patients with TE-SCC with CLM have better prognosis, which supports the current AJCC staging system for esophageal SCC.

## Background

Lymph node metastasis (LNM) is most common in esophageal squamous cell carcinoma. The bi-directional or skip node spread is a key feature of LNM in patients with thoracic esophageal squamous cell carcinoma (TE-SCC), with a metastasis rate of 23.4-49.5% in the cervical node [1–4].

In the past two decades, advances in esophageal cancer surgery have been remarkable. Radical esophagectomy with extensive lymphadenectomy in the mediastinum, abdomen, and neck (so-called three-field lymphadenectomy, 3FL) has been the mainstay treatment for TE-SCC. The surgical approach can sufficiently expose the surgical field and completely dissect related lymph nodes with metastasis [1–5].

According to the Guidelines for Clinical and Pathologic Studies on Carcinoma of the Esophagus issued by the Japanese Society for Esophageal Diseases, the cervical lymph nodes (LNs) were classified into 101 (paraesophageal nodes), 102 (deep cervical nodes), 103 (retropharyngeal LNs), and 104 (supraclavicular LNs) areas. Each area is divided into left and right parts [6]. In the seventh edition of the American Joint Committee on Cancer tumor node metastasis (AJCC TNM) staging system for esophageal squamous cell carcinoma issued in 2009, LNs from the neck to the abdomen are defined as regional LNs. In the sixth edition AJCC TNM staging system, the subdivision of “M” classification into M1A and M1B according to the presence of nonregional LN involvement is not longer used [5]. In addition, whether metastasis to the cervical LNs, especially supraclavicular LNs (104), should be classified as local or distant metastasis has not be proposed. In the present retrospective study, the prognostic factors were analyzed in 547 patients with TE-SCC with cervical LNM after receiving extended esophagectomy with 3FL.

## Methods

### Patient population

From January 1993 to March 2007, 1715 consecutive patients with biopsy-proven TE-SCC were treated with 3FL at the Fujian Province Cancer Hospital, Fujian Medical University, Fuzhou, Fujian, China. Medical records of these patients were retrieved. Patients meeting the following criteria were selected for this study: (1) pathologically confirmed as squamous cell carcinoma of the esophagus and underwent extended esophagectomy plus 3FL, (2) the number of dissected LNs was ≥15, (3) presurgical enhanced computed tomography scan did not reveal LN with a diameter >1 cm in the cervical area (including supraclavicular area), (4) did not undergo chemotherapy and radiotherapy before esophagectomy and did not undergo chemotherapy after esophagectomy, and (5) did not have distant metastasis. According to the seventh edition of the AJCC TNM staging system released in 2009, N is subclassified based on the number of positive regional LNs (N1, 1-2 positive LNs; N2, 3-6 positive LNs; and N3, ≥7 positive LNs) [5]. This study was performed in accordance with the Declaration of Helsinki and was approved by the ethics committee of Fujian Provincial Cancer Hospital. All patients provided written informed consent form for storage of their information in the hospital database and for using this information in this study. Of the 1715 patients, 547 patients were with cervical LNM, 296 patients underwent esophagectomy only, and 251 patients underwent radiotherapy after esophagectomy. The field of LNM was in accordance with the cervical, mediastinal, and abdominal LNs.

### Surgical procedures

The resection of the thoracic esophagus was performed through a cervical incision, a right thoracotomy, and a laparotomy. Details of the procedure were described elsewhere [1]. According to the guidelines for clinical and pathologic studies on carcinoma of the esophagus issued by the Japanese Society for Esophageal Diseases, the cervical LNs were classified into 101 (paraesophageal nodes), 102 (deep cervical nodes), 103 (retropharyngeal lymph nodes) and 104 (supraclavicular nodes) areas. Each area is divided into left and right parts [6].

### Radiotherapy

Patients underwent radiotherapy 3-4 weeks after esophagectomy. T-shaped fields were used. The T-shaped field included bilateral supraclavicular fossi, mediastinum, left gastric nodes, and the tumor bed. The medium total radiation dose consisted of 50 Gy for the tumor bed administered in 2 Gy of daily dose fractions, 5 fractions a week, over a period of 5 weeks [7].

### Follow-up

Patients were instructed to undergo follow-up evaluations every 3 months for the first year, every 6 months for the next 2 years, and annually thereafter. As of May 1, 2009, 90.1% of the patients returned for follow-up according to the schedule. Survival status of patients who did not come at the scheduled follow-up times was updated through telephone calls or letters every 6 months. Survival status of patients who could not be reached in this manner was obtained through the Fujian Public Safety Bureau’s registration center system. In total, 1336, 799, and 447 patients were followed up for 1, 3, and 5 years, respectively.

### Statistical analysis

Statistical analysis of group differences was performed using the Chi-square test for categorical variable data. Survival plots of patients were constructed using the Kaplan-Meier method and were compared using the log-rank test. A Cox regression proportional hazard multivariate analysis was performed to identify statistically significant factors associated with overall survival (OS). *P* < 0.05 was considered to be statistically significant. All statistical analyses were performed using the software package SPSS 15.0.

## Results

### Rate and pattern of LNM

In total, 547 of the 1715 patients met the inclusion criteria. The mean number of dissected LNs was 25.8 (range, 15-73). The frequency of any LNM was 31.9%. Specifically, the rates of cervical LNM for upper, middle, and lower TE-SCC were 44.2%, 31.5%, and 14.4%, respectively (*P* < 0.0001) (Table 1). The rates of LNM to 101, 104, 102, and 103 regions were 28.4%, 8.2%, 1.2%, and 0.2%, respectively. The rates of LNM from upper, middle, and lower TE-SCC to 101 and 104 were significantly different (*P* < 0.05) (Table 1). For patients with cervical metastasis, the rates of LNM to 101, 104, 102, and 103 regions were 89.0%, 25.6%, 3.7%, and 0.5%, respectively.

**Table 1:** **Characteristics of LNM in 1715 patients with TE-SCC**

	All patients	Location of esophageal tumor				
Variable		Upper	Middle	Lower	χ ^2^Value	***P-***value
Number of patients (%)	1715 (100)	274 (16.0)	1281 (74.7)	160 (9.3)		
Mean number of dissections						
Nodes per patient (range)	25.8 (15-73)	26.8 (15-68)	25.7 (15-71)	24.7 (15-73)		
Number of positive CLM (%)	547 (31.9)	121 (44.2)	403 (31.5)	23 (14.4)	41.698	<0.0001
Paraesophageal (101), n (%)	487 (28.4)	108 (39.4)	358 (27.9)	21 (13.1)	34.843	<0.0001
Deep cervical (102), n (%)	20 (1.2)	7 (2.6)	12 (0.9)	1 (0.6)	5.575	0.062
Retropharyngeal (103), n (%)	3 (0.2)	2 (0.7)	1 (0.1)	0 (0.0)	5.802	0.055
Supraclavicular (104), n (%)	140 (8.2)	31 (11.3)	104 (8.1)	5 (3.1)	9.049	0.011

*Abbreviations:* *CLM* cervical lymph node metastasis, *LNM* lymph node metastasis, *TE-SCC* thoracic esophageal squamous cell carcinoma.

### Relationship between cervical lymph node metastasis and survival

**Figure 1:**
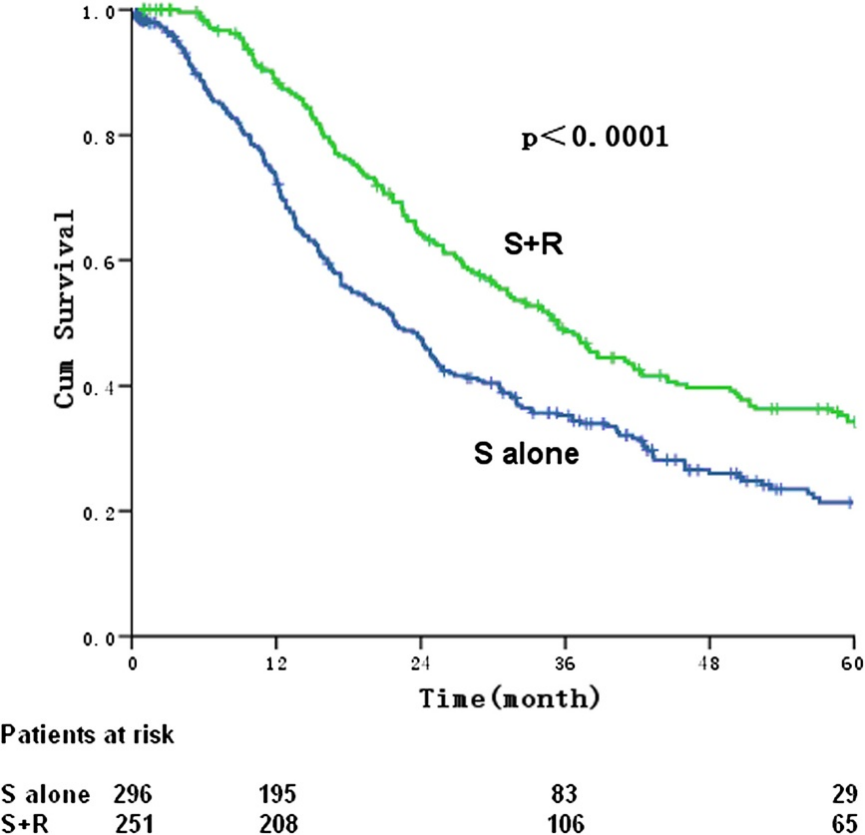
**Overall survival of patients who underwent surgery only (S, blue line) and who underwent surgery followed by radiation (S + R, green line) for thoracic esophageal squamous cell carcinoma.**

The 3-year and 5-year survival rates for patients (n = 547) with LNM were 41.5% and 27.7%, respectively. The median survival was 27.5 months. The 5-year survival rates and the median survival times were 21.3% versus 34.2%, and 21.9 months versus 35.4 months after surgery only (n = 296) versus surgery plus postoperative radiotherapy (n = 251), respectively [*P* < 0.0001 for both, hazard ratio (HR) (95% CI) 0.641 (0.521-0.788)] (Figure 1).

**Figure 2:**
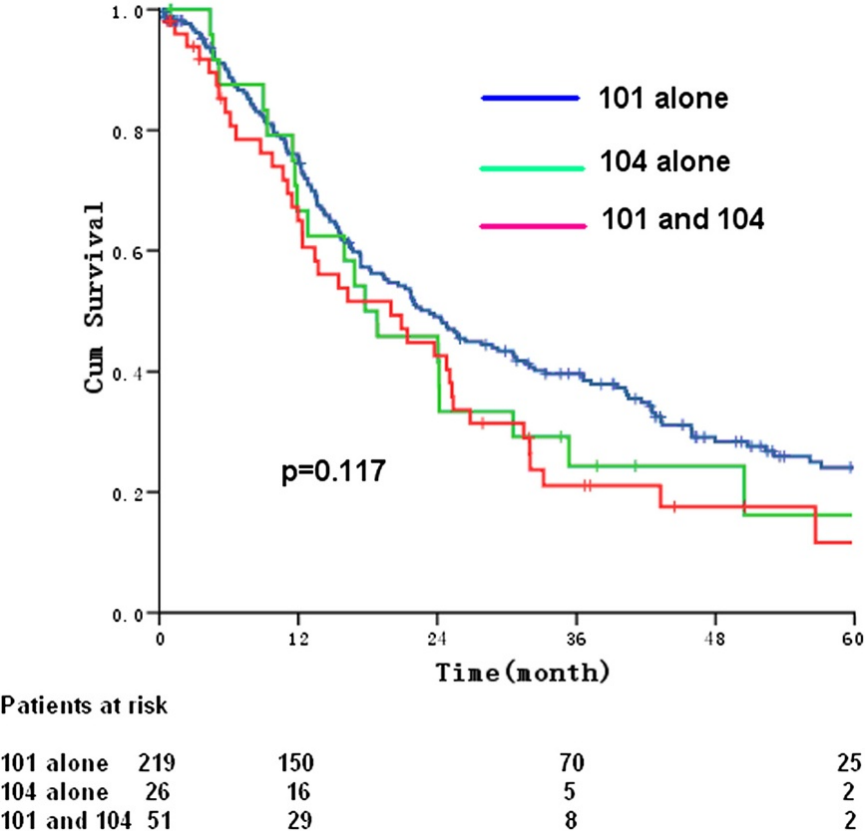
**Overall survival of patients presenting with positive nodes in the 104 region (green line), the 101 region (blue line), and in both (red line) regions.**

**Figure 3:**
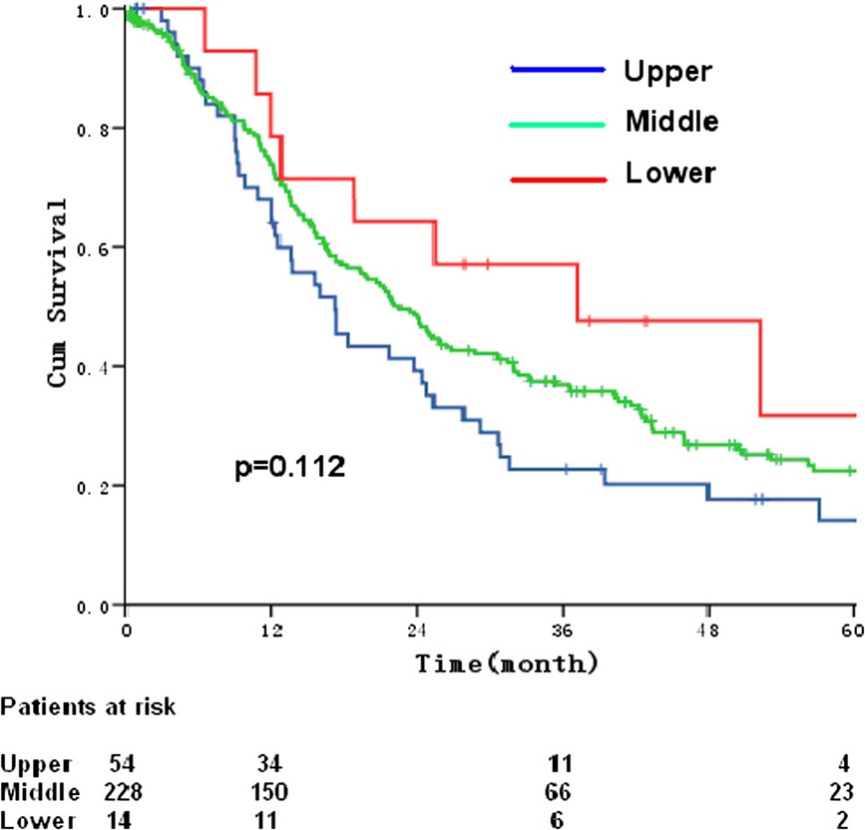
**Overall survival of patients presenting with positive nodes in the lower region (i.e., middle and lower mediastinal and upper abdominal beds) (red line), the upper region (i.e., cervical and upper mediastinal beds) (blue line), and in the middle region (green line).**

In surgery only group, the 5-year OS rates for patients’ metastasis to 101 LN alone, 104 LN alone or both 101 LN and 104 LN were 24.1%, 16.2%, and 11.7%, respectively. The median survival times were 23.3 months, 20.0 months, and 17.7 months, respectively [*P* = 0.117, HR (95% CI) 1.129 (0.996-1.280)] (Figure 2). The 5-year OS for patients with upper, middle, and lower TE-SCC were 17.7%, 22.5%, and 31.7%, respectively. The corresponding median survival times were 17.3 months, 22.6 months, and 37.2 months, respectively [*P* = 0.112, HR (95% CI) 0.734 (0.549-0.980)] (Figure 3).

### Analysis of prognostic factors of survival

Univariate analysis showed that sex, tumor length by x-ray, pT stage, pN stage, the number of fields with positive LNs, and treatment modality were predictors for survival. Age, tumor location, and histopathological type were not statistically significant predictors of survival (*P* > 0.05) (Table 2).

**Table 2:** **Univariate analysis of prognostic factors of survival in patients with TE-SCC with CLM**

		5-year	Median survival		
Variable	All (%)	Survival rate (%)	(Months)	χ ^2^value	***P-***value
Patients	547 (100)	27.7			
Sex				8.323	0.004
Male	406 (74.2)	24.6	24.8		
Female	141 (25.8)	37.0	39.5		
Age (years)				0.225	0.635
<60	335 (61.2)	27.8	25.9		
≥60	212 (38.8)	27.1	31.5		
Thoracic tumor location				0.456	0.796
Upper	121 (22.1)	31.7	29.2		
Middle	403 (73.7)	26.6	26.8		
Lower	23 (4.2)	23.3	25.5		
Differentiation				1.623	0.444
Low	118 (21.6)	23.4	24.1		
Intermediate	349 (63.8)	29.1	27.8		
High	80 (14.6)	28.6	28.3		
Tumor length (cm)				7.638	0.006
≤5	283 (51.7)	31.7	32.0		
>5	264 (48.3)	23.4	23.6		
pT stage				20.517	<0.0001
pT1	16 (2.9)	86.7	53.6		
pT2	84 (15.4)	41.6	43.4		
pT3	386 (70.6)	23.1	26.4		
pT4	61 (11.2)	23.2	22.5		
Number of nodal metastases				63.872	<0.0001
1-2	226 (41.3)	43.3	49.7		
3-6	221 (40.4)	20.3	23.5		
≥7	100 (18.3)	9.9	16.7		
Number of fields with positive lymph nodes^a^				55.313	<0.0001
1 field	191 (34.9)	43.0	43.3		
2 fields	214 (39.1)	25.5	29.2		
3 fields	142 (26.0)	10.2	19.3		
Treatment program				18.145	<0.0001
Surgery only	296 (54.1)	21.3	21.9		
Surgery + radiation	251 (45.9)	34.2	35.4		

*Abbreviations:* *CLM* cervical lymph node metastasis, *TE-SCC* thoracic esophageal squamous cell carcinoma.

^a^1 field (cervical lymph nodes), 2 fields (cervical + mediastinal, and/or cervical + abdominal lymph nodes), 3 fields (cervical + mediastinal + abdominal lymph nodes) with positive lymph nodes.

Multiple Cox regression indicated that sex, pT stage, pN stage, the number of fields with positive LNs, and treatment modality were independent predictors for survival (Table 3).

**Table 3:** **Multivariate analysis of prognostic factors of survival in patients with TE-SCC with CLM**

Variable	Regression coefficient B	SE	Wald value	HR (95% CI)	***P***-value
Sex (male vs. female)	-0.294	0.127	5.342	0.745 (0.581-0.956)	0.021
Tumor length (≤5 cm vs. >5 cm)	0.202	0.106	3.651	1.224 (0.995-1.505)	0.056
pT category (T1, 2, 3, 4)	0.283	0.096	8.687	1.327 (1.100-1.602)	0.003
Number of nodal metastases (1-2, 3-6, ≥7)	0.332	0.102	10.533	1.393 (1.140-1.702)	0.001
Fields of LNM (1 field, 2 fields, 3 fields)	0.203	0.100	4.109	1.225 (1.007-1.490)	0.043
Treatment program (surgery only vs. surgery + radiation)	-0.414	0.107	15.025	0.661 (0.536-0.815)	<0.0001

### Survival of different fields of positive lymph nodes according to the pN stage

**Figure 4:**
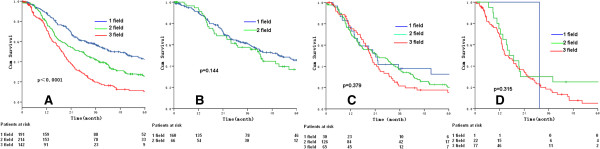
**The survival of different fields of positive lymph nodes according to the different pN stages as entire group (A), pN1 stage (B), pN2 stage (C), and pN3 stage (D).**

**Figure 5:**
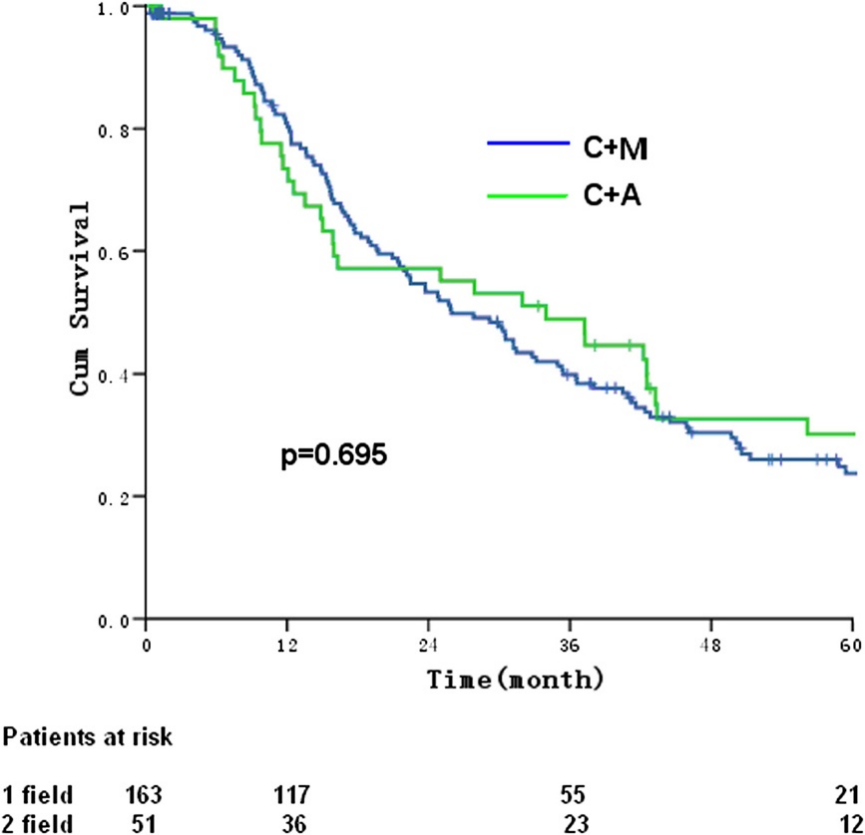
**The survival of patients with positive lymph node between cervical + mediastinal group and cervical + abdominal group.**

The 5-year OS rates were 43.0%, 25.5%, 10.2% in patients with 1 field (cervical LNs), 2 fields (cervical + mediastinal, and/or cervical + abdominal LNs), and 3 fields (cervical + mediastinal + abdominal LNs) positive LNs, respectively [*P* < 0.0001, HR (95% CI) 1.643 (1.437-1.878)] (Figure 4A). Subgroup analysis showed that the number of fields of positive LNs did not impact the OS according to different pN stage (all *P* > 0.05) (Table 4 and Figure 4B-D). The OS between cervical + mediastinal positive LNs and cervical + abdominal positive LNs were not significantly different (Table 4 and Figure 5).

**Table 4:** **Survival of different fields of positive lymph nodes according to the pN stage**

		5-year	Median survival		
Variable	All (%)	Survival rate (%)	Time (months)	χ ^2^value	***P***-value
pN1				2.136	0.144
1 field	160 (29.3)	45.6	51.8		
2 fields	66 (12.1)	36.8	42.3		
pN2				1.940	0.379
1 field	30 (5.5)	32.6	24.2		
2 fields	126 (23.0)	20.3	24.9		
3 fields	65 (11.9)	15.5	21.9		
pN3				2.311	0.315
1 field	1 (0.2)	0.0	31.5		
2 fields	22 (4.0)	25.0	16.7		
3 fields	77 (14.1)	5.1	14.7		
Fields of LNM				0.154	0.695
C + M	163 (76.2)	23.7	25.9		
C + A	51 (23.8)	30.1	34.0		

*Abbreviations:* A, abdominal; C, cervical; LNM, lymph node metastasis; M, mediastinal.

^a^1 field (cervical lymph nodes), 2 fields (cervical + mediastinal, and/or cervical + abdominal lymph nodes), 3 fields (cervical + mediastinal + abdominal lymph nodes) with positive lymph nodes.

*Abbreviations:* CI = confidence interval; CLM = cervical lymph node metastasis; HR = hazard ratio; LNM, lymph node metastasis; SE = standard error; TE-SCC = thoracic esophageal squamous cell carcinoma.

### Pattern of disease progression

Postoperative radiotherapy reduced the recurrence rate of cervical and mediastinal LN compared with surgery alone (*P* < 0.05). The pattern of disease progression in patients with and without postoperative radiotherapy is shown in Table 5.

**Table 5:** **Pattern of disease progression**

Variable	Surgery (n = 296) (%)	Surgery + postoperative radiotherapy (n = 251) (%)	χ ^2^value	***P***-value
Site of lymph node metastasis				
Cervical lymph nodes	42 (14.2)	13 (5.2)	12.192	<0.0001
Mediastinal lymph nodes	23 (7.8)	9 (3.6)	4.318	0.038
Abdominal lymph nodes	10 (3.4)	13 (5.2)	1.094	0.296
Tumor bed	6 (2.0)	2 (0.8)	1.426	0.2326
Distant metastasis	70 (23.6)	57 (22.7)	0.067	0.795
Locoregional and distant recurrence	126 (42.6)	93 (37.1)	1.721	0.190

### Toxicity of postoperative radiotherapy

Early toxicities related to postoperative radiotherapy were gastrointestinal reactions (swallowing pain and loss of appetite) accounting for 28.3% (71 patients), bronchitis (cough) accounting for 21.1% (53 patients), and leukopenia accounting for 34.3% (86 patients, including 80 patients with grade 1-2 and 6 patients with grade 3).

Late toxicities were nonmalignant pleural effusion pericardial accounting for 2.4% (6 patients), radiation-induced pulmonary fibrosis accounting for 2.0% (5 patients), thoracic ulcer bleeding accounting for 1.2% (3 patients), anastomotic stricture accounting for 1.6% (4 patients), and anastomotic fistula accounting for 0.4% (1 patient).

## Discussion

In the present study, pertinent results include that cervical LNM was the highest in patients with upper TE-SCC, followed by patients with middle and lower TE-SCC. Metastasis to paraesophageal nodes was most common. Metastasis to deep cervical nodes was less common. Metastasis to either retropharyngeal LNs or supraclavicular LNs was rare. The 5-year survival rates of patients undergoing surgery only were similar irrespective of whether there was metastasis to 101 LN alone, 104 LN alone, or both 101 LN and 104 LN. Multivariate factor analysis showed that the independent prognostic factors for survival were sex, pT stage, pN stage, the number of fields with positive LNs, and treatment modality. Cervical lymph node metastasis (CLM) was independent of tumor location.

There is controversy with regard to the prognostic significance and staging classification of cervical LNM in patients with TE-SCC. Most studies suggest that patients with cervical LNM have a better prognosis than those with hematogenous metastasis and thus cervical LNM should be included in “N” instead of “M” staging. Lerut *et al.* reported that the 5-year OS for patients with positive LNs was 27.2% after 3FL in patients with middle TE-SCC [8]. Fang *et al.* reported that 5-year OS for patients with positive cervical nodes was 20.0% after 3FL with TE-SCC [9]. Tachimori *et al.* reported that 3-year OS for patients with positive cervical nodes was 43.8% after 3FL with TE-SCC [10]. Hsu *et al.* enrolled 488 patients who underwent primary curative resection without neoadjuvant therapy for esophageal cancer between 1995 and 2006. They found the 3-year OS rate was 35.4%. The 3-year OS rate was equivalent among patients in N1 (23.3%), M1a (22.0%), and nonregional LNM-related M1b (18.5%). No survival difference was noted (18.5%). However, differences in survival rate were evident between patients with and without distant metastasis (*P* < 0.001) [11]. Kato *et al.* reported that in patients who underwent 3FL, the survival of patients with cervical LNM was significantly better than that of patients with hematogenous metastasis (*P* = 0.002). In patients without hematogenous metastases, the survival curve for the patients with histologic cervical LNM did not significantly differ from that of patients with mediastinal or abdominal LNM [12]. Rice *et al.* also found that the survivals were similar between patients in M0 classification and M1 classification (*P* < 0.0001). However, the survivals were significantly different between patients in M1a subclassification and M1b subclassification (*P* = 0.9) [13].

The results from the current study are similar to those reported by other researchers and support the current AJCC staging system which considers cervical LN to be regional LN [8–12]. The patients with cervical LN metastasis are classified as one group according to the AJCC staging system, and there is no explicit deliberation on whether the LNs adjacent to the cervical esophagus and supraclavicular LNs should be included. However, the cervical LN metastasis is classified elaborately into four groups including cervical esophageal LNs, cervical posterior deep LNs, retropharyngeal LNs, and supraclavicular LNs by the Japanese Society for Esophageal Diseases, though there was no published report on the prognosis related to this classification on cervical LNM. In the present study, the patients who underwent surgery only were classified into three groups, group of cervical esophageal LN metastasis, group of supraclavicular LN metastasis, and group of both cervical esophageal and supraclavicular LN metastasis. The stratified analysis on these three groups indicated that there was no significant difference in terms of 5-year survival rate, with the rate of 24.1%, 16.2%, and 11.7%, respectively (*P* = 0.117). These findings were in accordance with the concept defined by the AJCC staging system (seventh edition) that all cervical LN metastasis shall be regarded as one common regional LN metastasis.

In the present study, the 5-year survival rates in the postoperational radiotherapy group and surgery only group were 34.2% and 21.3%, respectively (*P* < 0.0001). The improvement in survival rate by postoperational radiotherapy might be due to blood vessels, lymphatic vessels, and surrounding organs, exposure of the lower cervical area is challenging during esophagectomy and complete removal of LNs is sometimes impossible, which will cause recurrence after surgery. Postoperative radiotherapy will reduce metastasis and increase survival [14].

It was widely believed that the number of fields of cervical LN metastasis was a vital factor for prognosis of thoracic esophageal carcinoma [13, 15], which was consistent with the results of the present study that the number of fields of cervical LN metastasis was an independent factor of prognosis. The further stratified analysis indicated that the number of fields of cervical LN metastasis and survival rate were not significantly different among the patients with different numbers of positive LNs (*P* > 0.05), and the possible underlying reason might be that the number of positive LNs is correlated to the number of fields of metastasis, implying that the number of positive LNs is the most critical factor for prognosis instead of number of fields of metastasis.

## Conclusion

This study demonstrates that patients with TE-SCC with cervical LNM have a better prognosis. Five-year survival in patients with TE-SCC with metastasis to paraesophageal nodes was similar to those with metastasis to supraclavicular LNs and supports the staging system of the current AJCC for esophageal squamous cell carcinoma that classifies cervical LN as regional LN. These patients will benefit from postoperative radiotherapy. Further perspective studies are needed to validate the conclusion.

## Abbreviations

3-FL: Three-field lymphadenectomy
AJCC: American Joint Committee on Cancer
CLM: Cervical lymph node metastasis
LN: Lymph node
LNM: Lymph node metastasis
TE-SCC: Thoracic esophageal squamous cell carcinoma
TNM: Tumor-nodes-metastasis.
